# Sand Fly–Associated Phlebovirus with Evidence of Neutralizing Antibodies in Humans, Kenya

**DOI:** 10.3201/eid2504.180750

**Published:** 2019-04

**Authors:** David P. Tchouassi, Marco Marklewitz, Edith Chepkorir, Florian Zirkel, Sheila B. Agha, Caroline C. Tigoi, Edith Koskei, Christian Drosten, Christian Borgemeister, Baldwyn Torto, Sandra Junglen, Rosemary Sang

**Affiliations:** International Centre of Insect Physiology and Ecology, Nairobi, Kenya (D.P. Tchouassi, E. Chepkorir, S.B. Agha, C.C. Tigoi, B. Torto, R. Sang);; Charité-Universitätsmedizin Berlin, Berlin, Germany (M. Marklewitz, F. Zirkel, C. Drosten, S. Junglen);; German Center for Infection Research, Berlin (M. Marklewitz, F. Zirkel, C. Drosten, S. Junglen);; Center for Virus Research, Kenya Medical Research Institute, Nairobi (E. Koskei, R. Sang);; University of Bonn, Bonn, Germany (C. Borgemeister)

**Keywords:** arbovirus, phlebovirus, sand flies, virus discovery, Kenya, viruses, neutralizing antibodies, Ntepes virus, Gabek Forest phlebovirus

## Abstract

We describe a novel virus, designated Ntepes virus (NPV), isolated from sand flies in Kenya. NPV has the characteristic phlebovirus trisegmented genome architecture and is related to, but distinct from, Gabek Forest phlebovirus. Diverse cell cultures derived from wildlife, livestock, and humans were susceptible to NPV, with pronounced permissiveness in swine and rodent cells. NPV infection of newborn mice caused rapid and fatal illness. Permissiveness for NPV replication in sand fly cells, but not mosquito cells, suggests a vector-specific adaptation. Specific neutralizing antibodies were found in 13.9% (26/187) of human serum samples taken at the site of isolation of NPV as well as a disparate site in northeastern Kenya, suggesting a wide distribution. We identify a novel human-infecting arbovirus and highlight the importance of rural areas in tropical Africa for arbovirus surveillance as well as extending arbovirus surveillance to include hematophagous arthropods other than mosquitoes.

Disease outbreaks caused by Zika, dengue, yellow fever, chikungunya, and Rift Valley fever viruses illustrate the threat posed by arthropod-borne viruses (arboviruses), which affect millions of patients worldwide each year (*1*–*3*). Major epidemic arboviruses are thought to have originated in tropical Africa, where they are known or thought to have caused local outbreaks before epidemic or pandemic spread. Early recognition of local outbreaks, including precise identification of the disease-causing agent, is key to effective preparedness against epidemics. However, active surveillance is poorly implemented in most seeding countries.

Monitoring programs for vectors of arboviral diseases often prioritize mosquitoes and ticks (*4*), neglecting other blood-feeding vectors, such as sand flies. Sand flies transmit protozoan parasites that cause leishmaniasis as well as phleboviruses (order *Bunyavirales*, family *Phenuiviridae*, genus *Phlebovirus*) (*5*,*6*). Sand fly–borne phlebovirus infections are known to occur in the Mediterranean basin (*7*,*8*). Some of these, such as sandfly fever Naples, Sicilian, and Toscana viruses, are of public health importance, especially Toscana virus, which causes meningitis and encephalitis in humans (*8*–*10*).

The International Committee for the Taxonomy of Viruses (ICTV) currently recognizes 10 viral species within the genus *Phlebovirus*: Rift Valley fever virus (RVFV), severe fever with thrombocytopenia syndrome (SFTS) virus, Uukuniemi phlebovirus, Bujaru phlebovirus, Candiru phlebovirus, Chilibre phlebovirus, Frijoles phlebovirus, Punta Toro phlebovirus, Salehabad phlebovirus, and sandfly fever Naples phlebovirus (*11*). The last 7 species are vectored by sand flies. Novel unclassified phleboviruses (e.g., Fermo, Granada, Punique, and Massilia viruses), as well as recently discovered flaviviruses in sand flies, underline the importance of these neglected vectors of arboviral diseases (*12*–*16*).

Sand fly–borne phleboviruses have been studied mainly in the Mediterranean region. Prevalence data for sub-Saharan Africa remain scarce. Studies have found serologic evidence of human infections with Karimabad, sandfly fever Naples, and sandfly fever Sicilian viruses in patients from Sudan with febrile illness (*7*,*17*,*18*). Human infections with sandfly fever Naples and Sicilian viruses have been reported in Uganda, Somalia, Djibouti, and Ethiopia (*7*,*19*). However, serologic surveys may be compounded by antigenic cross-reactivity between phleboviruses (except in neutralization assays), thereby precluding the unequivocal identification of the circulating sand fly–borne virus (*5*). Few studies have incorporated viral genetic characterization.

We describe a previously unknown phlebovirus discovered during vector surveillance in Kenya, which we designate Ntepes virus (NPV), after the place of sampling of virus-infected sand flies. The complete genome of NPV was sequenced, viral species tropism in cell culture assessed, and pathogenicity in vertebrates proven by infection of mice. Human serum samples from Ntepes and other communities yielded evidence of human infection based on specific virus neutralization.

## Methods

### Sandfly Trapping and Virus Isolation

**Figure 1:**
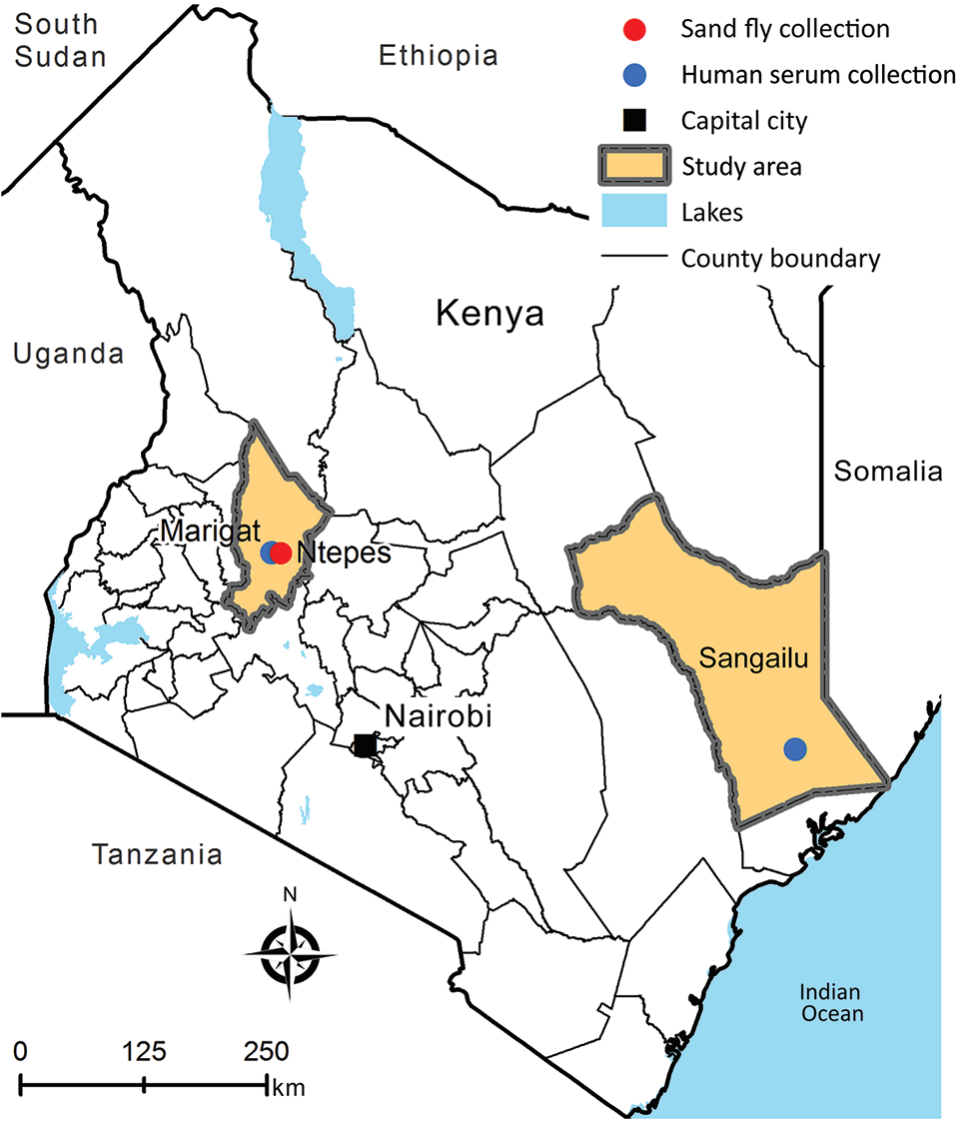
Geographic location of sand fly collection site (Ntepes) and district hospitals of Marigat and Sangailu, where human serum samples were collected, Kenya.

We trapped sandflies using CDC light traps (John W. Hock, https://johnwhock.com) in villages of the Marigat district, Baringo County, Kenya, in February 2014 (Figure 1). We homogenized pools of 5–50 female specimens in minimum essential medium (MEM), inoculated an aliquot of the clarified supernatant (50 μL) into Vero cells, and incubated it for up to 14 days with daily monitoring for cytopathic effect (CPE). We passaged CPE-positive supernatant onto fresh Vero E6/7 cells; virus stock solution was generated from the first passage and used for all further experiments. We determined the infectious titer by 50% tissue culture infectious dose (TCID_50_) assay using 10-fold serial dilutions from^–^ 10^–1^ to 10^–12^ of the virus stock inoculated in 5 wells each of subconfluent Vero cells seeded in a 96-well plate. We calculated the virus titer according to Reed and Muench (*20*). We estimated the minimum infection rate (MIR) in sandflies using the formula [number of positive pools/total specimens tested] × 1,000.

### Broad Reverse Transcription PCR Screening

We extracted RNA from cytopathic cell culture supernatant (200 μL aliquot) using the Viral RNA Mini Kit (QIAGEN, https://www.qiagen.com) and eluted it in 50 μL of buffer. We performed cDNA synthesis using SuperScript III reverse transcription (Invitrogen, https://www.thermofisher.com) and random hexamer primers (TIB Molbiol, https://www.tib-molbiol.com), followed by reverse transcription PCR (RT-PCR) for orthobunyaviruses, alphaviruses, and flaviviruses (*21*–*24*). We screened for sand fly–borne phleboviruses by targeting the RNA-dependent RNA polymerase (RdRp) gene using degenerate primers (*25*). We compared the nucleotide sequences to GenBank using blastn (https://blast.ncbi.nlm.nih.gov/Blast.cgi) and subjected them to initial phylogenetic analysis in MEGA6 (*26*). We further extracted RNA from pooled sand flies and tested it by real-time PCR using NPV-specific primers (forward, 5′-GCAAGAAAGCACTGTGGTGG; reverse, 5′-CGTATGATGATCGGCCACCA; probe, 5′-6-FAM-ACAGCCACCTCTGATGATGC-IBFQ). 

### Genotyping of Sand Flies and Blood Meal Analysis

We amplified the barcode region of the cytochrome *c* oxidase subunit I (*COI*) gene using published primers (*27*). We extracted genomic DNA from individual bloodfed sand fly specimens using the QIAGEN DNeasy Blood and Tissue Kit (QIAGEN). We amplified a 500-bp fragment of the 12S mitochondrial rRNA gene as described (*28*), sequenced the PCR products, and compared them to GenBank database data. We inferred species-level identification on the basis of ≥98% identity spanning >300 bp, as described by Valinsky et al. (*29*).

### Next-Generation Sequencing, Genome Annotation, and Phylogenetics

We purified and concentrated virions from the supernatant of infected Vero cells by ultracentrifugation through a 36% sucrose cushion. We extracted viral RNA using the QIAGEN RNeasy Kit according to the manufacturer’s instructions. We generated cDNA using the Maxima H Minus Double-Stranded cDNA Synthesis Kit and random hexamer primers (Thermo Fisher Scientific, https://www.thermofisher.com). We prepared DNA libraries using the Nextera XT DNA Sample Preparation Kit and analyzed them on an Illumina MiSeq instrument with the MiSeq Reagent Kit v3 (Illumina, https://www.illumina.com). We identified viral reads by reference mapping to phleboviruses as well as by BLAST comparisons against a local amino acid sequence library containing translations of open reading frames (ORFs) of phleboviruses. We closed sequence gaps by conventional RT-PCR followed by Sanger sequencing. We performed genome assembly using Geneious (http://www.geneious.com) and confirmed genome terminal sequences by rapid amplification of cDNA ends (RACE-PCR; Life Technologies, https://www.thermofisher.com). We identified ORFs using Geneious, compared nucleotide and amino acid sequences with other sequences by blastn and blastx searches against the GenBank database, and identified protein motifs by web-based comparison to the Pfam database (http://www.pfam.janelia.org). We identified putative transmembrane regions by prediction of the hydropathy profile using TMHMM (http://www.cbs.dtu.dk/services/TMHMM-2.0) and predicted N-linked glycosylation sites using the NetNGlyc 1.0 server (http://www.cbs.dtu.dk/services/NetNGlyc).

We aligned nucleotide and amino acid sequences of the ORFs of the respective genome segments with related viral sequences in Geneious using MAFFT (*30*). Phylogenetic trees were inferred by the maximum-likelihood (ML) method using the best suitable substitution matrix (LG) identified by Modeltest, as implemented in MEGA. We performed confidence testing based on 1,000 bootstrap iterations (*31*).

### In Vitro Viral Growth Kinetics

We infected cell lines from insects (LL-5, sand fly; C6/36, mosquito), humans (HEK293-T), small mammals (BHK-21, hamster; VeroE6/7, primate; MEF, mouse; EidNi, bat) and livestock (PK-15, swine; ZN-R, goat; DF-1, chicken; KN-R, cattle) in doublets, at a multiplicity of infection of 0.1. We harvested aliquots of infectious cell culture supernatants every 24 h for periods of 7 d and quantified viral genome copies by real-time RT-PCR with plasmid-based quantification standards.

### In Vivo Pathogenesis in Suckling Mice

We intracerebrally inoculated 100 μL of the viral stock of the first passage, as well as 3 consecutive 2-fold dilutions, into 3–4-day-old Swiss Albino suckling mice. The doses used in the experimental infection were quantified by plaque assays in Vero cells as described previously (*32*) and corresponded to viral titers of 4 × 10^6^, 2 × 10^6^, 1 × 10^6^, 5 × 10^5^, and 2.5 × 10^5^ PFU/mL. We included noninfectious MEM as a negative control. We observed all mice 2 times/day for up to 14 days for signs of disease. We homogenized brains from recently dead mice in 1 mL of cell culture media and plaque-titrated them on Vero cells.

### Human Serum Samples and Neutralization Tests

Archived serum samples from the Marigat district hospital, taken during 2010–2011, and from Sangailu Health Centre in the Hulugho subcounty in northeastern Kenya, collected during 2010–2012, were available (Figure 1). We performed a virus neutralization test using 2-fold serial dilutions of serum samples (1:20 to 1:640). We mixed 50 μL of the serial serum dilutions with 70 TCID_50_ of NPV. Mixtures were incubated at 37°C in the presence of 5% CO_2_ for 1 h, then used for infection of a confluent Vero E6/7 cell monolayer seeded in 96-well culture plates with 2 wells/dilution. After 7 days of incubation, we recorded the highest serum dilution at which no CPE was observed in at least 50% of the wells as the neutralization titer.

We tested NPV-reactive human serum samples with RVFV, Gabek Forest virus (GFV), and Karimabad virus (KARV) for serologic cross-reactivity, as described earlier in this article. In addition, we tested GFV- and KARV-positive serum samples with NPV in 2-fold serial dilutions from 1:5 to 1:20 (Table).

**Table:** NT reactivity with NPV, GFV, KARV, and RVFV of serum samples from febrile persons and healthy controls in Kenya*

Sample ID	Origin	Age, y/sex	Occupation	Acute febrile infection†	Reactivity against			
					NPV	RVFV	GBV	KARV
H01	Marigat	8/F	Student	Fever/chills, head/joint/muscle aches	1:40	None	None	None
H02	Marigat	10/F	Student	Fever/chills, cough, head/joint/muscle aches, eye pain, diarrhea	1:20	None	None	None
H03	Marigat	18/F	Student	Fever/chills, cough, head/joint/muscle aches, jaundice, abdominal pain	1:40	None	None	None
H04	Marigat	19/M	Shop attendant	Acute febrile illness—fever/chills, cough, head/muscle aches	1:20	None	None	None
H05	Marigat	29/M	Driver	Fever/chills, head/joint/muscle aches	1:80	None	None	None
H06	Sangailu	17/F	Housewife	Fever	1:40	None	None	None
H07	Sangailu	25/M	Herdsman	Fever/chills, headache, diarrhea	1:320	1:1,280	None	None
H08	Sangailu	42/F	Housewife	Fever/chills, headache, abdominal pain	1:40	None	None	None
H09	Sangailu	50/F	Housewife	Fever/chills, cough, head/joint/muscle aches	1:20	None	None	None
H10	Sangailu	24/M	Herder	Fever/chills, head/joint/muscle aches, diarrhea	1:320	None	None	None
H11	Sangailu	53/F	Housewife	Fever, cough, headache, abdominal pain, muscle ache	1:20	None	None	None
H12	Sangailu	26/M	Shepherd	Healthy control	1:20	None	None	None
H13	Sangailu	30/F	Housewife	Healthy control	1:160	None	None	None
H14	Sangailu	65/M	Pastoralist	Healthy control	1:40	1:320	None	None
H15	Sangailu	62/M	Herdsman	Healthy control	1:80	1:640	None	None
H16	Sangailu	34/F	Housewife	Healthy control	1:80	None	None	None
H17	Sangailu	52/F	Housewife	Healthy control	1:20	None	None	None
H18	Sangailu	50/M	Herdsman	Healthy control	None	1:1,280	None	None
H19	Sangailu	57/M	Herdsman	Healthy control	None	1:320	None	None
H20	Sangailu	16/M	Herder	Fever/chills, head/joint/muscle aches, abdominal pain	1:160	1:160	None	None
H21	Sangailu	18/M	Student	Fever, cough, headache, abdominal pains	1:80	None	None	None
H22	Sangailu	9/F	Student	Fever/chills, cough, abdominal pains, joint/muscle aches	1:160	None	None	None
H23	Sangailu	19/F	Housewife	Fever/chills, headache, abdominal pains	1:40	None	None	None
H24	Sangailu	30/F	Housewife	Fever, head/joint/muscle aches, abdominal pain	1:160	1:40	None	None
H25	Sangailu	17/M	Herder	Fever/chills, head/joint/muscle aches, abdominal pain	1:160	None	None	None
H26	Marigat	17/M	Student	Fever/chills, head/joint/muscle aches	1:40	None	None	None

*GFV, Gabek Forest virus; ID, identification; KARV, Karimabad virus; NPV, Ntepes virus; NT, neutralizing test; RVFV, Rift Valley fever virus. †Fever defined as body temperature >38°C.

### Ethics Considerations

Approval for the study was granted by the Scientific and Ethical Review Unit and Animal Care and Use Committee of the Kenya Medical Research Institute (SSC Protocol nos. 1560 and KEMRI/SERU/CVR/003/3312). All animal experiments were carried out in accordance with the regulations and guidelines of the Kenya Medical Research Institute.

### GenBank Accession Numbers

The NPV genome was deposited in GenBank under accession nos. MF695810–MF695812. The *COI* sequence obtained from the virus-positive sand fly pool was deposited in GenBank under accession no. MG913288.

## Results

### NPV Isolation and Characterization 

In total, 6,434 sand flies were trapped (Figure 1). A subset of 5,481 sandflies was pooled and the resulting 111 pools individually inoculated in VeroE6/7 cells. One pool consisting of 8 females induced CPE 4–5 days postinfection. Sequence analysis of the *COI* gene of the sand flies of this CPE-positive pool suggested that sand flies were of the genus *Sergentomyia*. We identified blood-meal hosts for 62 blood-fed specimens sampled at the same place and time as the pooled specimens. Results revealed that 56 (90.3%) had fed on humans, 2 (3.2%) on snakes, and 1 (1.6%) each on a frog, lizard, cow, and ostrich. The infectious cell culture supernatant tested negative for RVFV, orthobunyaviruses, alphaviruses, and members of genus *Flavivirus*. We amplified a 0.5-kb fragment of the RdRp gene of sand fly–borne phleboviruses using degenerated primers (*25*). The sequence showed the highest pairwise identity of 79% to GFV and 75% to KARV. We sampled a subset of 953 individual sand fly samples 2 years after the initial study and tested it in pools of 10 by specific RT-PCR for the cultured virus; results were negative.

**Figure 2:**
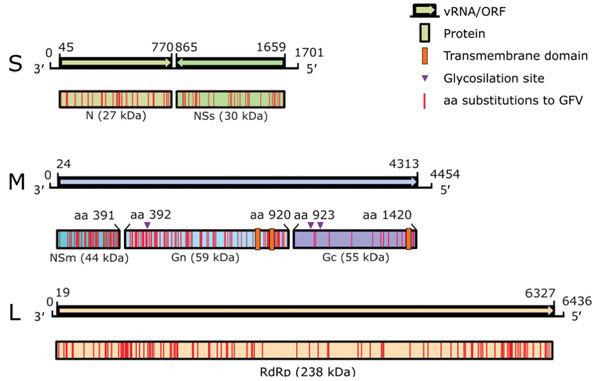
Genome organization of novel sand fly–associated phlebovirus Ntepes virus identified in Kenya. Sequence length of the L, M, and S segments (in bp) and encoded predicted proteins RdRp, Gn, Gc, N, and nonstructural proteins NSm and NSs (in kDa) are indicated; ORF positions (length in bp) are also indicated. GFV, Gabek Forest virus; L, large segment (encoding the RdRp protein); M, medium segment (encoding the nonstructural protein NSm and the 2 glycoproteins Gn and Gc); N, nucleocapsid protein; ORF, open reading frame; RdRp, RNA-dependent RNA polymerase; S, small segment (encoding the N protein and nonstructural protein NSs in an ambisense manner); vRNA, virus RNA.

Analysis of the complete genome by next-generation sequencing confirmed isolation of a novel phlebovirus. The virus was tentatively termed Ntepes virus, after the location where the sand flies were collected. The virus exhibits the characteristic tripartite-segmented genome organization of phleboviruses, comprising the large (L) segment, which encodes the RdRp protein; the medium (M) segment, encoding a glycoprotein precursor protein (GPC) that is posttranslationally cleaved into 2 viral surface glycoproteins (Gn and Gc) and a nonstructural protein (NSm); and the small (S) segment, encoding the nucleocapsid (N) protein and a nonstructural protein (NS) in an ambisense manner (Figure 2). Highest sequence similarities to GFV were 93% to RdRp, 88% to GPC, 79% to Nsm, 85% to N, and 90% to NS. NPV has the typical conserved genome termini shared among phleboviruses (5′-ACACAAAG and CUUUGUGU-3′) (*8*).

**Figure 3:**
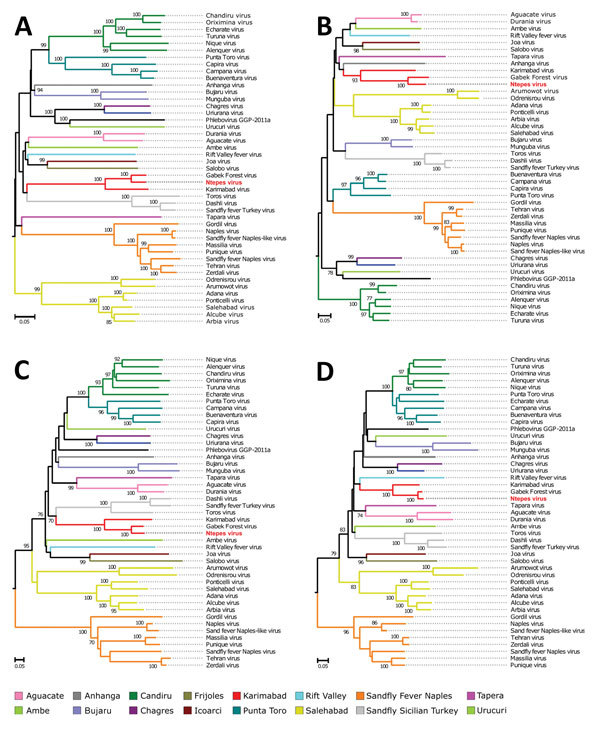
Phylogenetic relationship of novel sand fly–associated phlebovirus Ntepes virus from Kenya (red bold text) in relation to other selected members of the *Phlebovirus* genus. A) RNA-dependent RNA polymerase; B) nucleocapsid protein; C) glycoprotein Gn; D) glycoprotein Gc. The phylogenetic trees were inferred based on complete large, medium, and small protein sequences, applying maximum likelihood analysis in PhyML version 3.0 (http://www.atgc-montpellier.fr/phyml/versions.php) using the LG substitution model. Statistical support of the tree topology was evaluated by bootstrap resampling of the sequences 1,000 times. Sequences are identified by virus name and branch colors. Bootstrap values >70 are indicated at the nodes. Scale bar represents numbers of substitutions per site.

Phylogenetic analyses of NPV RdRp, Gn, Gc, and N proteins and all available sand fly–borne phlebovirus sequences indicate that NPV forms a strongly supported clade with GFV and KARV. NPV branches as a sister taxon to GFV in all genes, suggesting NPV to be a member of the Karimabad species complex (Figure 3). However, the designation of the Karimabad species complex is not yet officially approved by the ICTV. For a provisional genetic classification, we analyzed the intragenetic distances among established phlebovirus species and unclassified isolates based on the RdRp gene. Pairwise nucleotide and amino acid distances between established species ranged from 38% to 62% for nucleotide distances and 39% to 68% for amino acid distances (Appendix Figure). For example, amino acid distance between Punta Toro virus and Candiru virus was 39% and between SFTS virus and sandfly fever Naples virus was 68%. Pairwise nucleotide distances ranged from 20% to 59% and amino acid distances from 6% to 69% when unclassified tentative species and variants pertaining to established species were included. For example, amino acid distance between Ponticelli virus and Adana virus was 6% and between Naples virus and SFTS virus was 69% (Appendix Figure). NPV showed 7% amino acid distance to GFV and 19% amino acid distance to KARV.

**Figure 4:**
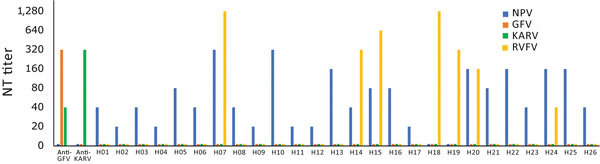
Neutralizing activity of novel sand fly–associated phlebovirus Ntepes virus from Kenya in relation to other selected members of the *Phlebovirus* genus. Anti-GFV and anti-KARV samples were tested along with 26 human serum samples. GFV, Gabek Forest virus; H, human; KARV, Karimabad virus; NPV, Ntepes virus; NT, neutralizing test; RVFV, Rift Valley fever virus.

Classical criteria for species demarcation in phleboviruses are based on serology, with established species showing at least 4-fold differences in 2-way neutralization tests (*11*). We confirmed that NPV did not react with antiserum against its next closest relatives, GFV and KARV, in neutralization tests (Figure 4). NPV Gn protein was 13% different and Gc 4% different from GFV. The Gn protein of phleboviruses is the key component for neutralization and is recognized by specific neutralizing antibodies (*33*).

Although sequence-based species demarcation criteria have not been determined for phlebovirus species, such criteria exist for the related orthobunyaviruses. Species demarcation criteria are now based on the RdRp gene, which shows >6% difference to the closest related virus. Previously unique orthobunyavirus species were defined on >10% difference in N protein sequences (*11*). The N proteins of NPV and GFV differ by 15% (GFV itself is not formally classified as a species, and any of the formally classified phlebovirus species are markedly more distant from NPV in this and other genes; Appendix Figure). We conclude, upon cumulative evidence, that NPV constitutes a putative novel species within the phlebovirus genus.

### Permissiveness in Vertebrates

**Figure 5:**
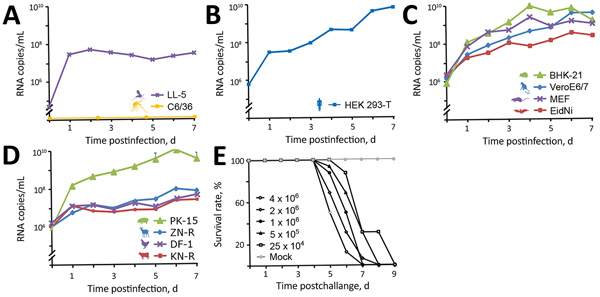
In vitro growth kinetics of novel sand fly–associated phlebovirus Ntepes virus from Kenya in different cell lines. A) Insects: LL-5, sand fly; C6/36, mosquito. B) Human: HEK293-T. C) Peridomestic wildlife: hamster, BHK-21; primate, VeroE6/7; mouse, MEF; bat, EidNi. D) Livestock: swine, PK-15; goat, ZN-R; chicken, DF-1; cattle, KN-R. Cells were infected with a multiplicity of infection of 0.1; supernatants were collected every 24 h for 7 d postinfection. Viral genome copies were measured at indicated timepoints by real-time reverse transcription PCR. E) Pathogenicity of Ntepes virus infection in mice. Litters of 2-day-old Swiss Albino suckling mice (8 mice/litter) were intracerebrally inoculated using the indicated virus titers or cell culture media as a control. Animals were monitored daily for signs of disease. Titers are shown in PFU/mL.

To obtain initial data on permissiveness, we performed in vitro growth analyses in a broad range of cell lines derived from different insect species (sand fly and mosquito), peridomestic wildlife (rodent, nonhuman primate, and bat), and livestock (swine, goat, chicken, and cattle) species, as well as from humans. Results revealed a broad susceptibility to NPV, with peak genome copy numbers in cells derived from swine and rodents (Figure 5, panel A). Cells derived from sand flies but not from mosquitoes were permissive, despite using C6/36 mosquito cells that are normally broadly susceptible to arboviruses because of a defect in their antiviral RNA interference response (*34*). These findings suggest a host range for NPV similar to those of KARV and GFV, which are transmitted by sand flies and infect rodents (*35*). It is not known whether rodents are amplificatory or dead-end hosts.

Because GFV is known to induce fatal disease in laboratory mice (*36*,*37*), we explored similarities in pathogenicity with NPV. We intracranially inoculated 3–4-day-old Swiss Albino suckling mice, causing tremors, hind-limb paralysis, prostration, and death 5–8 days postinfection (Figure 5, panel B). Time to death was clearly correlated with virus dose. All animals had high infectious virus concentrations in the brain (mean 2.9 × 10^6^ PFU/mL). Taken together, the in vitro and in vivo pathogenicity studies of NPV, including the pathogenicity in suckling mice, may suggest that rodents and sand flies may be involved in the maintenance cycle of NPV.

### Evidence for Human Infection with NPV

To test whether NPV infects humans, we analyzed 187 archived serum samples: 59 samples from the Marigat district hospital in the area where NPV-infected sand flies were trapped, and 128 samples collected in northeastern Kenya at Sangailu Health Centre (Figure 1). All patients from Marigat, as well as 98 patients from Sangailu, had symptoms compatible with acute infectious diseases. The remaining 30 samples from Sangailu came from healthy controls.

Twenty-six (13.9%) serum samples neutralized NPV, with titers ranging from 1:20 to 1:320 (Figure 4). Women and men were infected at equal rates. Positive samples originated from Marigat (10.2%) and Sangailu (15.6%), without statistical differences in rates (Fisher exact test odds ratio [OR] 0.6, 95% CI 0.19–1.70; p = 0.37). Detection rates in Sangailu did not differ between healthy and febrile patients (Fisher exact test OR 1.4, 95% CI 0.40–4.3; p = 0.58) (Table; Figure 4). No NPV nucleic acids were detected in serum samples by NPV-specific RT-PCR, suggesting no causative link to the present symptoms with NPV. The detection of NPV neutralizing antibodies in geographically unlinked regions of Kenya suggests widespread previous human exposure and infection.

Because NPV is genetically most closely related to GFV and KARV, we tested all NPV-positive serum samples for ability to cross-neutralize GFV or KARV. All tests yielded negative results, providing further support for the classification of NPV as a separate serotype (and species). Because RVFV frequently causes outbreaks in East Africa, we also tested against RVFV, which, according to its phylogenetic relationship with NPV, is not expected to cross-react with NPV. Seven of 26 NPV-neutralizing serum samples were also reactive with RVFV, showing titers that did not correlate in height with titers against NPV (Table; Figure 4). Absence of correlation of titers suggests previous RVFV infection rather than cross-reactivity between RVFV and NPV.

## Discussion

We identified a high percentage of neutralizing antibodies to NPV in humans living in the NPV-endemic area by neutralization assay, confirming that NPV represents a distinct phleboviral species that causes infection in humans. The fact that the virus was isolated through an exploratory sampling effort is an indicator of the existence of undetected and uncharacterized viruses in this part of Kenya. Although mosquitoes have been the focus of studies on emerging arboviruses, the discovery of a novel sand fly–borne phlebovirus with evidence for human exposure across Kenya indicates the need to broaden vector surveillance activities.

Toscana, sandfly fever Sicilian, and sandfly fever Naples viruses are distributed in the Mediterranean region and northern Africa. GFV has been reported from Sudan, Senegal, Central African Republic, Nigeria, and Benin (*38*). KARV occurs in eastern and central Asia (*7*,*39*,*40*), as well as Sudan, Egypt, and Nigeria (*7*). According to this geographic distribution, GFV seems to be the most likely sand fly–borne phlebovirus to co-occur in Kenya. Our results show that NPV-immune serum samples do not react with GFV or KARV, suggesting that the reactivity of the positive human samples was the result of previous infection with NPV.

NPV in Kenya may occupy a niche that is taken by GFV and KARV in northern Africa or eastern and central Asia. Several characteristics of NPV suggest parallels between the host ranges of NPV and GFV. GFV has been detected in rodents (*38*) but has been detected in arthropods in only a single study in sand flies (*35*). Further, the virus was shown to be able to infect *Phlebotomus* species under laboratory conditions (Tesh R. Studies of the biology of phleboviruses in sand flies. Paper presented at Yale University School of Medicine, New Haven, CT, USA, 1983), suggesting that GFV is maintained in a transmission cycle that involves rodents and sand flies and that it occasionally infects humans (*35*). NPV was isolated from sand flies and replicates in vitro in sand fly–derived cell lines but not in mosquito cells, similar to sandfly Sicilian and Naples viruses (*41*). Infection studies with cell lines derived from livestock and peridomestic wildlife species showed that NPV replicates ≈10–100 times better in rodent and swine cell lines than in cells derived from other animals, suggesting the involvement of rodents or swine as potential amplificatory hosts for NPV. 

*COI* gene analyses from the virus-positive sand fly pool suggests that species of the genus *Sergentomyia* have been infected with NPV. Blood-meal analyses revealed that 90% of the analyzed blood-fed sand flies had fed on humans, confirming a likely role as vectors of NPV to humans. Our findings provide new evidence that *Sergentomyia* flies do not strictly feed on reptiles but also feed frequently on humans (*42*,*43*).

The NPV antibody prevalence rate in humans (13.9%) is comparable to that of GFV, which is 17%–60% in Sudan, 3%–10% in Egypt, and 3% in Nigeria (*7*). KARV antibody prevalence is 1%–11% in Sudan, 2% in Egypt, and 1%–62% in regions of Iran and Russia (*7*). Human serum samples from northern Kenya have been tested and yielded no antibodies against GFV or KARV, which matches our results (*7*). 

NPV appears to have a wide distribution in Kenya; we found equal exposure rates in 2 geographic sets of humans sampled >600 km apart. The serum samples from this study were collected during 2010–2012, suggesting that NPV has been present in humans since at least 2010. Sand fly pools collected in 2014 had low infection rates (MIR 0.18, 1/111 pools, 5–50 sand flies/pool), possibly resulting from collection during a period with low transmission rates. The estimated MIR is lower compared with previous sand fly infections with phleboviruses such as Punique (MIR 6.7) (*14*), Massilia (MIR 3.7) (*12*), and Toscana (MIR 2.2) viruses (*44*), although comparable to Toros (MIR 0.26) and Zerdali (MIR 0.35) viruses (*45*). The significance of just 1 isolate of the novel phlebovirus from 111 sand fly pools may seem limited, but it is noteworthy that circulation of RVFV, a phlebovirus with huge epidemic potential, is generally detected at low rates in vectors during interepidemic periods. For instance, multiple surveillance efforts sampling and analyzing thousands of primary and secondary RVFV vectors from outbreak hotspot areas failed to yield any RVFV isolates (*46*,*47*), yet RVFV infection rates in mosquitoes during the 2006–2007 outbreak in Kenya were high, ranging between 0.8 and 10.65 per 1,000 for primary vectors (*2*). 

The outcome of infection experiments in mice suggests that NPV could cause diseases such as GFV and RVFV infection (*36*,*37*). The neglect of sand fly–borne phleboviruses in Africa is exemplified by outbreaks of acute febrile illness associated with sandfly fever Sicilian virus in Ethiopia, which, for a long time, had remained misdiagnosed as malaria (*48*), as well as an outbreak of febrile illness probably associated with sandfly fever Naples virus in Sudan (*17*).

The symptoms reported among most of the tested patients in this study cannot be conclusively linked to NPV infection, as indicated by antibodies in symptomatic patients and healthy controls, demanding further studies of possible disease association. Clinical studies using specific real-time PCR are necessary to detect viral RNA in humans and to measure the clinical impact of NPV.

Appendix Distance matrix and distance plots of phleboviruses used in the study of sand fly–associated phleboviruses, Kenya.
