# Supplementary material for: Sand Fly–Associated Phlebovirus with Evidence of Neutralizing Antibodies in Humans, Kenya
Source: Emerg Infect Dis. 2019 Apr;25(4):681–90. doi: 10.3201/eid2504.180750 (PMC6433041; doi:10.3201/eid2504.180750)
Supplement: Appendix — Distance matrix and distance plots of phleboviruses used in the study of sand fly–associated phleboviruses, Kenya. [file 18-0750-Techapp-s1.pdf]

Article DOI: https://doi.org/10.3201/eid2504.180750

Sand Fly–Associated Phlebovirus with Evidence of Neutralizing Antibodies in Humans, Kenya

Appendix

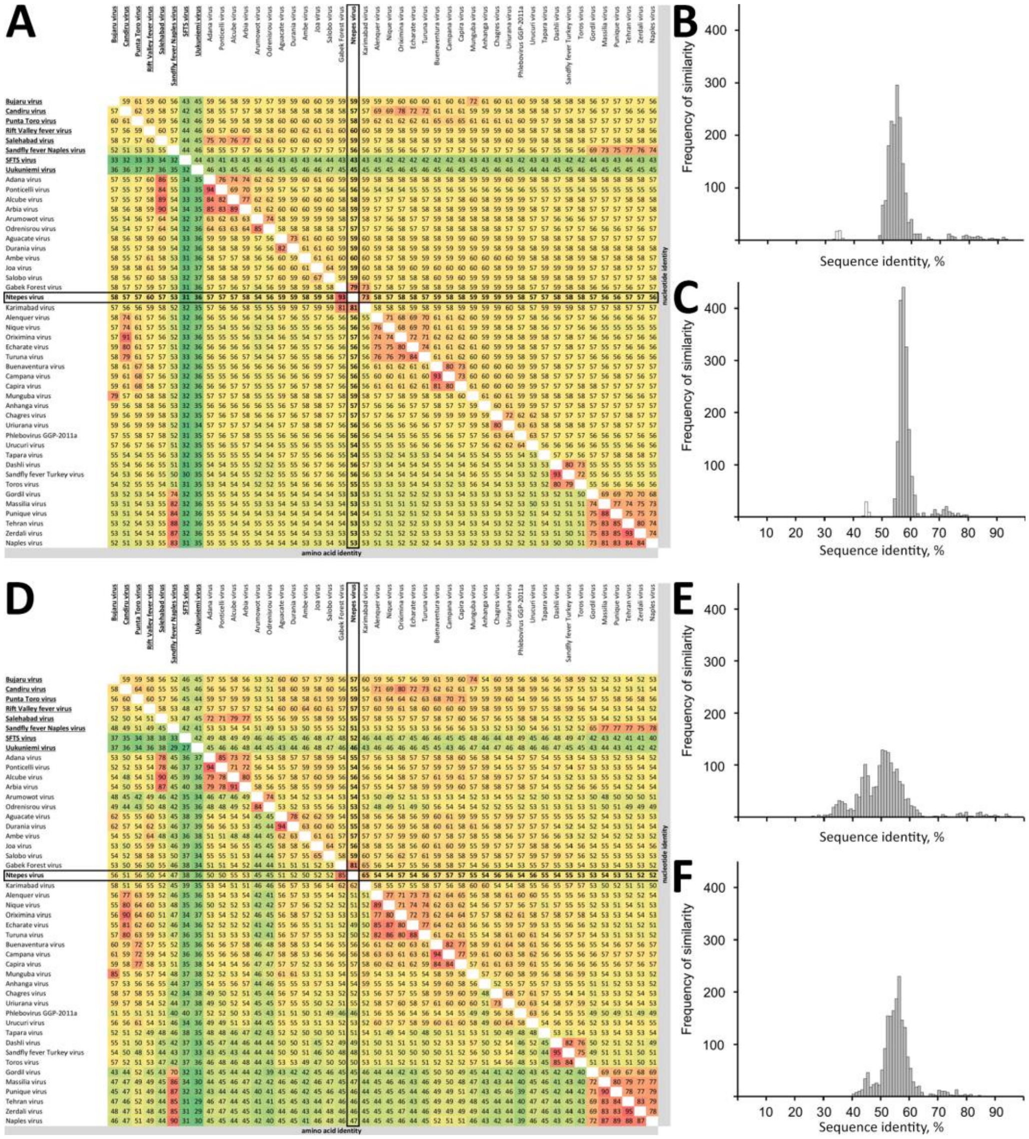

Appendix Figure. Distance matrix and distance plots of phleboviruses. Pairwise interspecies identities among complete RdRp genes (A) and nucleoprotein genes (D) based on nucleotide sequences are shown on the upper right side and pairwise interspecies identities based on transduced amino acid

sequences on the lower left. Colors indicate level of identity from high (red), medium (yellow), to low (green). Established species according to the International Committee for the Taxonomy of Viruses are underlined. The frequency of pairwise amino acid (B, E) and nucleotide (C, F) identities among complete RdRp genes and nucleoprotein genes is plotted.
